# Crosswell electromagnetic modeling from impulsive source: Optimization strategy for dispersion suppression in convolutional perfectly matched layer

**DOI:** 10.1038/srep32613

**Published:** 2016-09-02

**Authors:** Sinan Fang, Heping Pan, Ting Du, Ahmed Amara Konaté, Chengxiang Deng, Zhen Qin, Bo Guo, Ling Peng, Huolin Ma, Gang Li, Feng Zhou

**Affiliations:** 1 Institute of Geophysics and Geomatics, China University of Geosciences, Wuhan, China; 2 Hubei coal geological survey institution, China National Administration of Coal Geology, Wuhan, China

## Abstract

This study applied the finite-difference time-domain (FDTD) method to forward modeling of the low-frequency crosswell electromagnetic (EM) method. Specifically, we implemented impulse sources and convolutional perfectly matched layer (CPML). In the process to strengthen CPML, we observed that some dispersion was induced by the real stretch *κ*, together with an angular variation of the phase velocity of the transverse electric plane wave; the conclusion was that this dispersion was positively related to the real stretch and was little affected by grid interval. To suppress the dispersion in the CPML, we first derived the analytical solution for the radiation field of the magneto-dipole impulse source in the time domain. Then, a numerical simulation of CPML absorption with high-frequency pulses qualitatively amplified the dispersion laws through wave field snapshots. A numerical simulation using low-frequency pulses suggested an optimal parameter strategy for CPML from the established criteria. Based on its physical nature, the CPML method of simply warping space-time was predicted to be a promising approach to achieve ideal absorption, although it was still difficult to entirely remove the dispersion.

To achieve an ultimate prospecting distance of 900 m^1^, the operating frequency of the crosswell electromagnetic (EM) method is usually applied at 5–1000 Hz and the scale of a crosswell target is usually below 10 m. These conditions result in a large boundary condition requirement in crosswell EM numerical simulation. We adopted the well-known finite-difference time-domain (FDTD) method with convolutional perfectly matched layer (CPML) to solve the forward modeling problem for low frequencies and small areas.

Based on the classical perfectly matched layer (PML)^2^,^3^, a complex frequency-shifted perfectly matched layer (CFS-PML)^4^ with strict causality was proposed. The CFS-PML can be set up near the target to decrease the number of grid cells and the memory required for efficiency in geophysical modeling. This process is easily performed in lossy, dispersive, or anisotropic media. The most critical advantage of the crosswell EM is that CFS-PML can maintain a weak late time reflection and powerfully absorb electromagnetic waves of a certain frequency^5^, which is pivotal in low-frequency modeling and has been successfully verified^6^. As a mature and efficient scheme to implement CFS-PML, CPML resolved the convolution between the complex frequency-shifted stretching function and the spatial derivatives of the magnetic and electric fields, which creates heavy computational complexity^7^,^8^.

Even though CPML was derived from elegant formulations, reality does not present such ideal conditions. The advantages of CPML noted above were not optimal in low-frequency modeling^9^, and the lack of optimality was suspected to produce numerical dispersion that gave rise to grid noise^10^,^11^. Because the second-order FDTD method suffered from anomalous numerical effects arising from numerical dispersion, high-order finite-difference and pseudo-spectral Maxwell solvers were proposed to effectively reduce discretization^12^. However, unfortunately, when the method based on high-order algorithms was implemented in CFS-PML, it was found that high-order PML exhibited absorption performance similar to that of the 2^nd^-order PML^13^. Moreover, in contrast with the traditional recursive convolution (RC) method to evaluate recursion, piecewise linear recursive convolution (PLRC)^14^ and trapezoidal recursive convolution (TRC)^15^ methods were proven to be more accurate, as was the case for high-order PMLs. If a high-order accuracy algorithm is the best solution for numerical dispersion in CPML or if the key to promoting the efficiency of CPML is to suppress numerical dispersion, the answer most likely resides in the absorption mechanism of CPML.

Research on the dominant absorption frequency of CPML has been in depth and widely applied^5^,^12^, whereas the research on numerical dispersion in CPML has only just begun^10^; this research has theoretically deduced the restrictions of grid interval in the linear dimension but has neglected the stretched coordinate interspaces in three-dimensional (3D) CPML. In this fundamental study on the crosswell EM, we focus on the origin, presentation and restriction of dispersion in 3D CPML.

## Methods

First, this section introduces the role and evaluation of pivotal CPML parameters that enable the absorption results to satisfy crosswell EM requirements. Then, we deduce and reveal the inevitable dispersion in outer CPML that prevents CPML from being ideal; as a result, the endeavor to promote CPML is given a theoretical basis.

### Pivotal parameters in CPML

Theoretically, CPML is based on stretched-coordinate Maxwell equations and the no-reflection situation that plane waves propagate in any stratified medium according to the constraint law of constitutive parameters; the purpose is the absorption of electromagnetic waves by the parameters *α* (degrees of freedom of the stretching factor), *σ* (conductivity) and *κ* (real stretch). The key parameters of CPML are summarized in Table 1.

In the Maxwell equations considering the stretching factors in CFS-PML and eliminating sources in the frequency domain, the Laplace operators in 3D Euclidean space are rewritten as general types. For example, the *S*_*ez*_ (the stretching factor in the Z direction and on the electric field component) can be described as follows^4^:


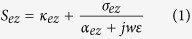


where *α*_*ez*_ is the degrees of freedom of the stretching factor in the Z direction and on the electric field, *σ*_*ez*_ is the conductivity in the Z direction and on the electric field in CFS-PML, *κ*_*ez*_ is the real stretch in the electric field’s Z direction^16^, and *ε* is the permittivity of the background medium.

After the Maxwell equations are transformed into the time domain, the CPML algorithm arises from the convolution of stretching factors. Eq. (2) represents the dispersive form of electric field *E*_*x*_ in the X direction in the time domain^17^ and can be generally applied in both the inner modeling space and the outer absorbing boundary:


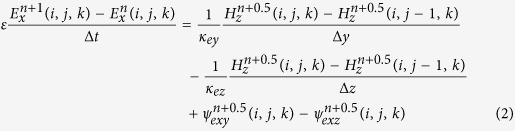


where Δ*t* denotes the time interval, Δ*y* and Δ*z* are the space intervals in the Y and Z directions, and the points in time are expressed as the superscripts of the electric field (*E*_*x*_), magnetic field (*H*_*z*_, *H*_*y*_) and convolution terms (*ψ*_*exy*_, 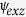).

The updating principle of the CPML algorithm is as follows: the real stretch *κ*_*ez*_ in the Z direction and on the electric field component is only renewed in CPMLs that are perpendicular to the Z coordinate, and the real stretch *κ*_*ey*_ in the Y direction and on the electric field component is only renewed in CPMLs that are perpendicular to the Y coordinate; their values remain 1 in other cases. 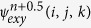 and 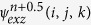 are the discrete convolutional terms. 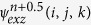 is only renewed in CPMLs that are perpendicular to the Z coordinate, and 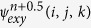 is only renewed in CPMLs that are perpendicular to the Y coordinate; their values remain 0 in other cases. Take the renewed equation of 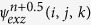 as an example:


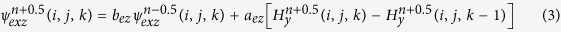


where the interim parameters *a*_*ez*_ and *b*_*ez*_ are as follows:


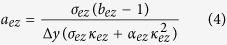



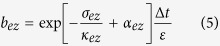


The renewed equations of *E*_*x*_ (Eqs (2), (5), 10, 11) can be extended to other components of the electromagnetic field. On the basis of the conventional renewed equations of FDTD^17^, CPML innovates two parameters^5^: *α* and *κ*. This research investigates the dispersion caused by *κ*, from which we acquire the optimal parameters for CPML.

Referring to Eq. (1), the characteristic frequency of a CPML in the Z direction can be described as follows^5^:


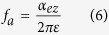


If *κ*_*ez*_ = 1, then only *α*_*ez*_ and *α*_*mz*_ (the degrees of freedom of the stretching factor in the Z direction and on the magnetic field) differentiate CPML and PML. When *f* >> *f*_*a*_ (*f* is the frequency of the incident wave), *α*_*ez*_ in Eq. (1) can be neglected, which means that the efficiency of the CPML-absorbing high-frequency incident wave is close to that of PML^6^. When *f* << *f*_*a*_, *S*_*ez*_ in Eq. (1) should be real and it is beyond the ability of the CPML to absorb low-frequency incident waves at that moment^5^. Therefore, the characteristic frequency *f*_*a*_ determined by *α*_*ez*_ is regarded to be the dominant absorption frequency of the CPML.

Restricted by the updating principle of 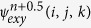 in Eq. (3), conventional CPMLs change their *α* only in a direction perpendicular to the perfect electric conductor (PEC) layer. For example, *α*_*ez*_ changes linearly from *α*_*inn*_ (the value of *α*_*ez*_ in the innermost boundary) to *α*_*out*_ (the value of *α*_*ez*_ in the outermost boundary) to ensure that the incident wave of a particular bandwidth is absorbed homogeneously^17^.


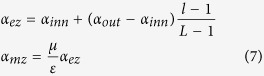


where *l* is the grid number from the innermost boundary to the current boundary and *L* is the number of boundary layers. The first step to set up the parameters of a CPML is to determine *α*_*inn*_ and *α*_*out*_ with Eq. (6) and then calculate all *α* using Equation (7).

*σ*_*ez*_ (the conductivity in the Z direction) and *σ*_*mz*_ (the permeability in the Z direction) directly affect the absorption efficiency of electromagnetic waves in CPMLs. Small *σ*_*ez*_ and *σ*_*mz*_ will lead to strong reflection by the PEC, whereas overlarge *σ*_*ez*_ and *σ*_*mz*_ will generate apparent induction fields in CPMLs^5^. Considering the zero reflection condition on the layer interface of a PML, *σ*_*ez*_ and *σ*_*mz*_ in the direction perpendicular to a PEC layer best promote absorption efficiency according to the exponential distribution between the background and the appropriate value:


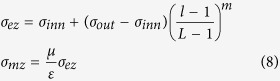


where *m* is the exponential power, generally from 2 to 4; *σ*_*inn*_ (conductivity of the innermost CPML) is equal to the background conductivity; and *σ*_*out*_ (conductivity of the outermost CPML) is *ξ* times that of *σ*_*inn*_ (coefficient *ξ* < 10). After we determine the assignments of *σ*_*ez*_ and *σ*_*mz*_, the choice of *σ*_*out*_ affects the pivotal role of the absorption efficiency of the CPML.

### Additional κ impacts on dispersion of the CPML

Greater attention to *κ*_*ez*_ and *κ*_*mz*_ (the real stretch in the Z direction and on the magnetic field) in the CPML is required. The present visualized understanding is that *κ*_*ez*_ and *κ*_*mz*_ bring about stretched coordinates of grids and contribute the same as conductivity: the larger the values of *κ*_*ez*_ and *κ*_*mz*_, the more intense the absorption efficiency. In forward experiments, if *κ*_*ez*_ and *κ*_*mz*_ are larger than a certain value, there will be a continuous reflected wave whose waveform is distorted and whose amplitude is even greater than that of the incident wave. This is the typical characteristic of dispersion^10^. Based on this discovery, we assumed that *κ*_*ez*_ and *κ*_*mz*_ will cause dispersion in CPML grids. Therefore, this section deduces a theory following the dispersion and then analyzes the anisotropy of phase velocity from difference approximations.

*κ*_*ez*_ is described by Eq. (9) in the Z direction perpendicular to the PEC layer^17^:


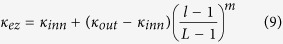


where *κ*_*inn*_ = 1 (the real stretch value of the innermost CPML) and *κ*_*out*_ is usually 5–11 (the real stretch value of the outermost CPML)^18^.

In accordance with *α*_*ez*_ in Eq. (7) and *σ*_*ez*_ in Eq. (8), *κ*_*ez*_ in Eq. (9) exponentially increases in a direction perpendicular to the PEC layer. In other words, these pivotal parameters are isotropic when parallel with the PEC plane. The following equations on dispersion in a 3D CPML can be simplified to 2D. If the CPML is perpendicular to the Y coordinate and the incident TE wave from any angle is perpendicular to the Z coordinate, then the electric vector is a transverse field to the Z coordinate. This is the so called “TEz pattern,” whose plane wave solutions in free space are as follows:


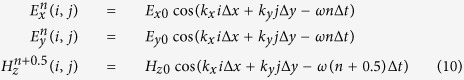


Equation (2) then leads to the 2D discrete Maxwell equation of the TEz pattern in the CPML, as shown in Eq. (11). The values of *κ*_*ez*_ and *κ*_*mz*_ are 1, whereas convolution terms 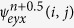 and 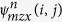 are 0 because of the CPML being perpendicular to the Y coordinate. Consequently, Eq. (11) is shown as a simplified result:


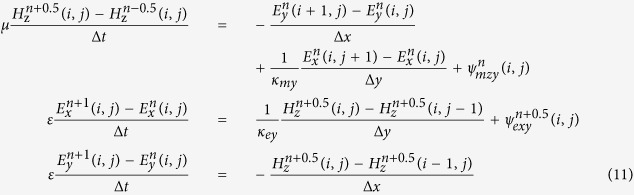


Then, we insert the plane wave solution of the TEz pattern (Eq. (10)) into Eq. (11) and combine it with the trigonometric identity:


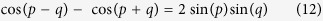


After *E*_*x*_, *E*_*y*_ and *H*_*z*_ are removed with this step, the dispersion in the 2D CPML is revealed in Eq. (13). Iteration of the convolution element 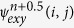 is based on the updating principles in Eq. (3) and is valued according to Eqs (7, 8, 9); *b*_*ez*_ in Eq. (4) and *a*_*ez*_ in Eq. (5) are always less than 0.01. *a*_*ez*_ << 1/*κ*_*ez*_Δ*z* and *b*_*ez*_ ≈ 0 relative to the renewed Eq. (2) of FDTD. Therefore, 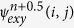 and 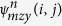 in Eq. (11) can mostly be ignored. Strictly speaking, 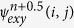 and 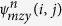 remain 0 before the incident wave arrives; however, the dispersion is an inherent attribution of the CPML and the iteration of the convolution is feasible based on these considerations. Eq. (13) is the final simplified result from dispersion in the 2D CPML when *κ*^2^ = *κ*_*ez*_*κ*_*mz*_:


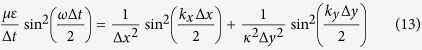


Because of the influence of *κ*, the dispersions of CPML and FDTD are different. For the sake of a visual description of such dispersions, the conventional uniform grids are unified as Δ*x* = Δ*y* = *δ* (*δ* represents the cube grid interval) and wave vectors satisfy Eq. (14):


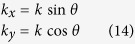


where *θ* is the angle between the Y coordinate and the propagation direction of the TEz pattern plane wave in the CPML. Substituting Eq. (14) into Eq. (13) results in the following:


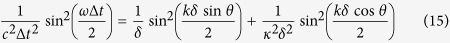


Because *k* = *ω*/*v*_*φ*_ = 2*π*/*λ* (*λ* is the wave length of the incident wave) and Δ*t* meets the Courant stability condition, it is reasonable that 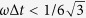 Eq. (15) is approximated by the following:


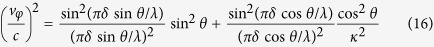


**Figure 1:**
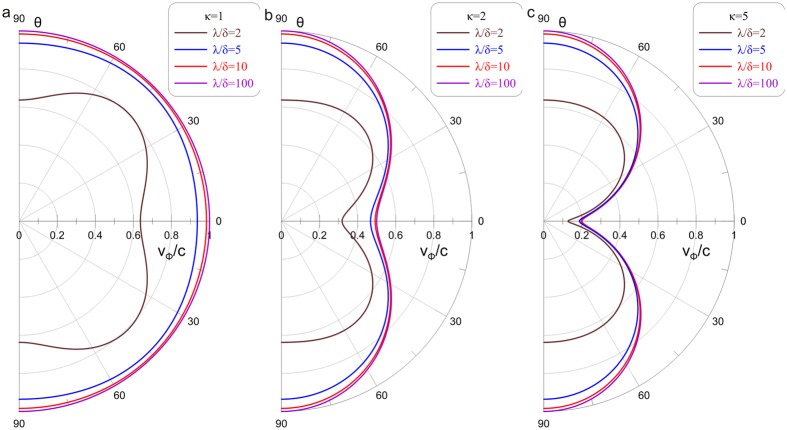
The anisotropy of *v*_*φ*_/*c* after difference approximations in a CPML. **(a)** For *κ* = 1, the relationship among *θ*, *λ*/*δ* and *v*_*φ*_/*c*. **(b)** For *κ* = 2, the relationship among *θ*, *λ*/*δ* and *v*_*φ*_/*c*. (**c)** For *κ* = 5, the relationship among *θ*, *λ*/*δ* and *v*_*φ*_/*c*.

Equation (16) means that *v*_*φ*_ (the phase velocity of TEz pattern plane wave) is relevant to *θ*, *λ*/*δ* and *κ* in the CPML. This relationship can be interpreted using three two-dimensional polar plots (see Fig. 1). One interpretation of Fig. 1 is that CPMLs are perpendicular to *θ* = 0°, the incident angle of the plane wave is *θ* from the arc, and the 4 curves represent *v*_*φ*_/*c* (the ratio of the plane wave’s phase velocity and the velocity of light) corresponding to different *λ*/*δ*. Three representative cases are listed: (a) *κ* = 1, (b) *κ* = 2, (c) *κ* = 5. The smaller the distance between the center and the curve, the smaller the phase velocity corresponding to *θ*, *λ*/*δ* and *κ*, which means that the dispersion in CPML is more severe.

When *κ* = 1, Fig. 1(a) shows the dispersion rule in conventional grids; the dispersion in uniform grids rises significantly until *λ*/*δ* increases to 10 and the dispersions in the directions of *θ* = 0° and *θ* = 90° are most intense. Although *κ* increases in Fig. 1(b,c), it is still the case that *v*_*φ*_ decreases when *λ*/*δ* decreases, and the dispersion is invariant compared with *κ* = 1 in the direction of *θ* = 90°. However, the grids’ dispersion increases relatively in Fig. 1(a), and *v*_*φ*_ decreases as *κ* increases, especially in the direction of *θ* = 0°, which means that the *v*_*φ*_ values differ greatly from *v*_*φ*_ in uniform grids, and *v*_*φ*_/*c* < 1/*κ* exists no matter how much *λ*/*δ* increases. Therefore, the dispersion in the CPML mainly occurs in the outer layers, where *κ* is proximal to *κ*_*out*_, as well as in the direction perpendicular to the PEC layer.

**Figure 2:**
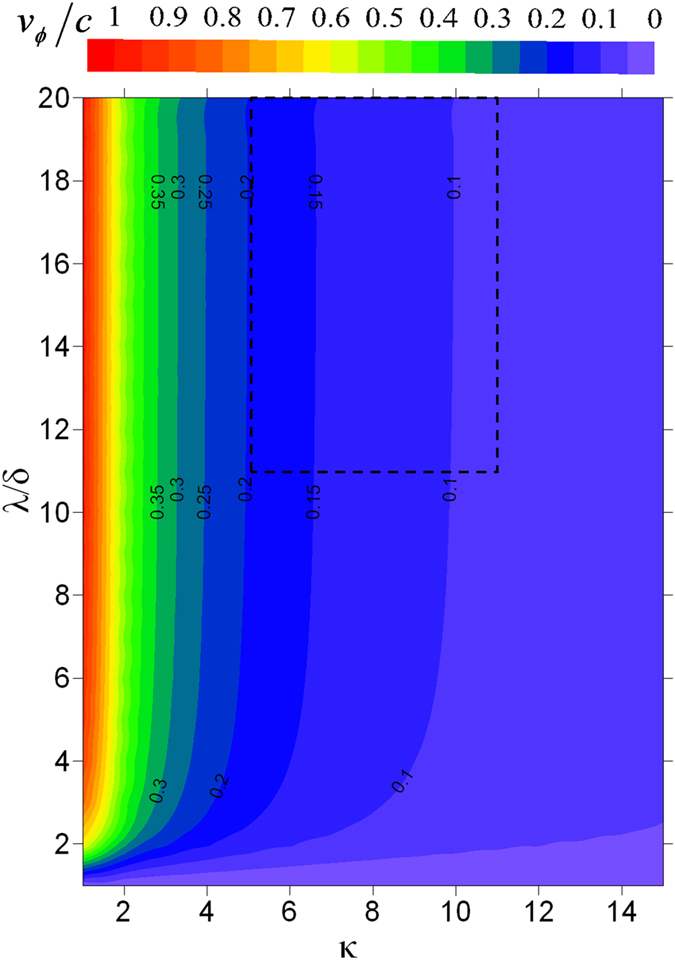
The relationship among *κ*, *λ*/*δ* and *v*_*φ*_/*c*.

In the attempt to suppress the grids’ dispersion, our biggest concern is the simulation region where *v*_*φ*_/*c* is undersized and serious dispersion is generated. To highlight the influence of the CPML parameter settings on the grid dispersion, Fig. 2 illustrates the relationship among *κ*, *λ*/*δ* and *v*_*φ*_/*c* from Eq. (16) when *θ* = 0°. The dispersion is merely tolerable near *κ* = 1, and it is invalid to simply reduce the grid spacing to suppress the dispersion of the CPML after *κ* > 1. However, the values of *κ* and *λ*/*δ* in conventional CPMLs (inside the dashed box) lead to *v*_*φ*_/*c* < 0.2 and the grids’ dispersion is too serious to ignore.

Because the increase in *κ* results in the increase in both absorption efficiency and dispersion, the assignment of *κ*_*out*_ should give consideration to both. If the dominant frequency of an electromagnetic source is relatively high, the skin effect will be strong. In this case, solely depending on *σ* is sufficient for absorbing electromagnetic waves and *κ*_*out*_ should be small so that the dispersion is negligible. If the dominant frequency of the electromagnetic source is relatively low, the absorption efficiency from *σ* will decrease simultaneously. Nonetheless, *κ*_*out*_ with large values may offset this shortage.

## Results

Because the dispersion is severe in conventional CPML, we should take actions to suppress it without sacrificing too much absorption efficiency. CPML forward experiments show that it is more complex to evaluate wave fields under relatively low-frequency conditions than under relatively high-frequency conditions. In this section, a frequency with an electromagnetic wavelength less than the forward boundary is defined as “relatively high-frequency,” whereas electromagnetic wavelengths greater than that are referred to as “relatively low-frequency.” This section focuses on simulation accuracy and optimal parameter setting to overcome the dispersion in the CPML. From relatively high-frequency modeling and snapshots, we verify the preceding conclusions and develop a qualitative understanding of dispersion suppression. Then, after the relatively low-frequency experiments are accomplished with an established set of criteria for absorption, we confirm optimal parameter settings from statistical results of abundant modeling. However, the analytical solution of a magnetic dipole pulse must first be solved as a foundation.

### The analytical solution of a magnetic dipole pulse

To analyze the absorption mechanisms of CPMLs, it is necessary to exclude medium influences during forward modeling; therefore, it is more dialectical to deduce the analytical solution in a vacuum.

Research on the crosswell EM commonly adopts magnetic dipole sources in a time-harmonic field^19^,^20^,^21^. In this research, the pulsed field with a magnetic dipole source is introduced into low-frequency crosswell EM modeling for enhancing transmit power^1^. This pulsed field makes the receiving coils observe the primary field during pulse emission and the secondary field after pulse stop, thereby improving the exploration efficiency of the crosswell EM. Thus far, analytical solutions of electromagnetic fields have been primarily deduced from time-harmonic dipole sources and step-field dipole sources; however, research on analytical solutions of magnetic dipole pulses has not been well developed. We start with the analytical solution in the time domain.

In spherical coordinates of free space, there is a normal component of a radiation field in the plane where a magnetic dipole source is located^22^:


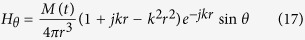


where *M*(*t*) is the magnetic dipole moment, *r* is the distance between receiving and transmitting coil, and *θ* is the angle between the normal direction of the receiving coil and the line through the receiving and transmitting coil. Substituting the wave number *k* = *ω*/*c* into Eq. (17) gives the following:


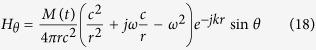


Eq. (18) is then converted from the frequency domain to the time domain as follows:


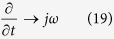



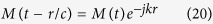


Finally, we achieve the radiation field equation of a magnetic dipole pulse in the time domain:


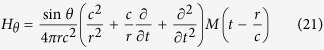


The analytical solution at the receiving coil is combined with *M*(*t*) (the formula of a magnetic dipole pulse) and Eq. (21) and is used in the following sections.

### The dispersion law from relatively high-frequency modeling experiments

In this section, a series of CPML absorption experiments is carried out in a vacuum with relatively high-frequency pulses. Because the dispersion is confirmed when using conventional CPML, it is indispensable to improve CPML parameter settings for qualitative understanding.

The regulation of *κ*_*ez*_ from Eq. (9) indicates that dispersion occurs mainly in the outer layers of a CPML and barely in the inner layers where the value of *v*_*φ*_/*c* is approximately 1. As long as the absorption boundary condition guarantees that the electromagnetic waves reflecting back into the inner space eventually are within *γ* (an acceptable proportion), the requirements for dispersion in a CPML are not as strict as those in an inner space using FDTD. It is pivotal that high-frequency waves from the outer layers of the CPML are absorbed by the inner layers of the CPML; as a consequence, *α*_*inn*_ with a large value in Eq. (7) has a certain level of suppression of dispersion.

**Figure 3:**
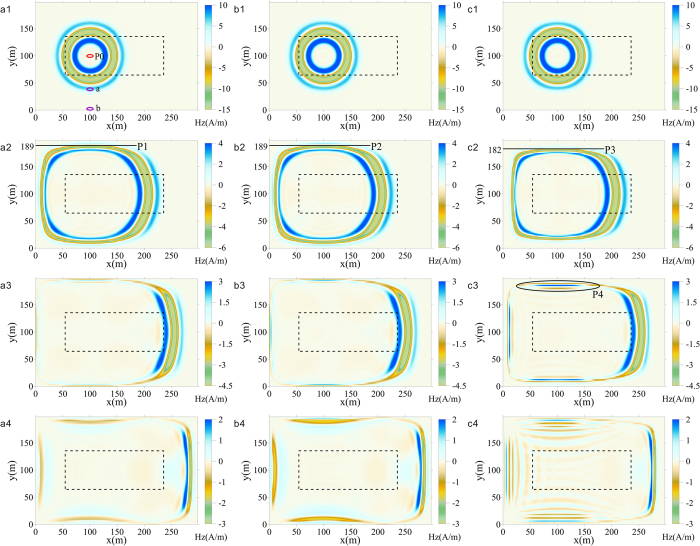
Snapshots of absorption effects in CPMLs at the same depth as the emission source. Under the condition that other parameters remain constant, the parameter settings of **(a1)–(a4)** are *κ*_*out*_ = 5, *α*_*inn*_ = 8.4 × 10^−4^ and *α*_*out*_ = 2.8 × 10^−4^; the parameter settings of **(b1)–(b4)** are *κ*_*out*_ = 5, *α*_*inn*_ = 2.8 × 10^−4^ and *α*_*out*_ = 8.4 × 10^−4^; and the parameter settings of **(c1)–(c4)** are *κ*_*out*_ = 11, *α*_*inn*_ = 8.4 × 10^−4^ and *α*_*out*_ = 2.8 × 10^−4^. The time of **(a1)–(c1)** is 0.48 × 10^−6^ s; the time of **(a2)–(c2)** is 0.74 × 10^−6^ s; the time of **(a3)–(c3)** is 1.11 × 10^−6^ s; and the time of **(a4)–(c4)** is 1.39 × 10^−6^ s.

**Figure 4:**
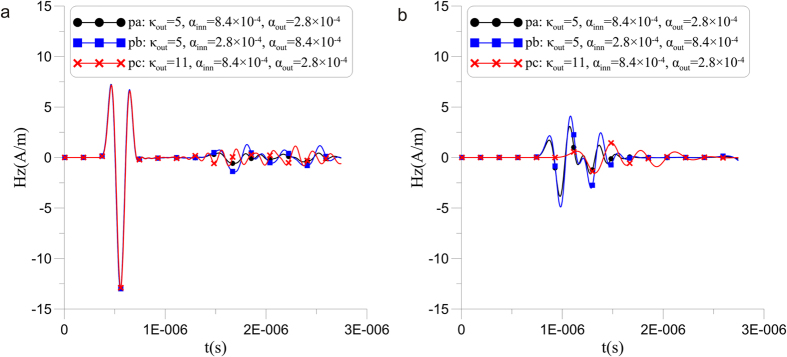
Hz variation over time with three parameter settings given as pa, pb and pc. Corresponding to Fig. 2 (a1), (a) is the forward solution in (100,40) and **(b)** is the forward solution in (100,4); both are in the CPML.

To verify the existence of dispersion and the feasibility of parameter setting in a CPML, numerical simulation experiments in a 3D vacuum were implemented. As shown in Fig. 3(a1), a magnetic dipole source transmits a Gauss pulse in the position p0 inside the modeling region. The maximum frequency of the source is close to the frequency that satisfies numerical dispersion conditions in a uniform grid. This scheme highlights the impact of dispersion from stretching grids. 32 CPML layers and 1 PEC layer were implemented outside of the dashed boxes in Fig. 3 as the absorbing boundary (2 m grid spacing) to amplify the variation of electromagnetic wave phase velocity in the CPML. Based on the dominant frequency of the pulse source (2 × 10^7^ Hz) whose pulsating wave length is less than 60 m, *α*_*inn*_ and *α*_*out*_ were calculated from Eq. (6); therefore, we can compare the inhibition results from possible *α*_*inn*_ and *α*_*out*_ (such as pa and pb in Fig. 4).

Figure 3 displays snapshot aggregations of Hz (the Z component in the magnetic field) combining 3 groups of parameter settings (pa, pb, pc) and 4 moments. The XY plane of the snapshots is perpendicular to the Z coordinate in a vacuum and at the same depth as the transmitting coil. The color bar indicates the values of Hz. The parameter settings of pa are *κ*_*out*_ = 5, *α*_*inn*_ = 8.4 × 10^−4^ and *α*_*out*_ = 2.8 × 10^−4^; the parameter settings of pb are *κ*_*out*_ = 5, *α*_*inn*_ = 2.8 × 10^−4^ and *α*_*out*_ = 8.4 × 10^−4^; and the parameter settings of pc are *κ*_*out*_ = 11, *α*_*inn*_ = 8.4 × 10^−4^ and *α*_*out*_ = 2.8 × 10^−4^. Parameter settings pa and pb show the absorption effect of high-frequency dispersion waves with different *α*_*inn*_ and *α*_*out*_. Parameter settings pb and pc show the appearance of dispersion from different *κ*_*out*_.

Figure 3(a1–c1) displays the moment that the Gauss pulse has been transmitted and electromagnetic waves have just entered the inner CPML, where there is no obvious reflection from the absorption boundary. The wave field characteristics for these three different parameter settings are almost the same, and the wave fronts of Hz are circular, which indicates that the dispersion is negligible in the internal forward area with a uniform grid.

For the next moment in time (see Fig. 3(a2–c2)), the wave fronts of Hz have entered the outer CPML. The shape of the wave front has transformed from rounded to rectangular; this phenomenon is reasonably explained by the relationship between *θ* and *v*_*φ*_ in Fig. 1(c). Moreover, the waveform in the CPML of Fig. 3(c2) is significantly different from those in Fig. 3(a2,b2). The lines p1, p2 and p3 indicate that the coordinates of the head wave and p1 and p2 are closer to the PEC than p3, which indicates that the propagation velocity of the electromagnetic wave is slower in Fig. 3(c2). This effect is demonstrated in Fig. 4(b), which includes the time-domain curves of Hz at a position 4 m from the PEC (such as circle b in Fig. 4). The time when the head wave arrives shows that the propagation velocity in conditions pa and pb are the same, although faster than in pc. In other words, the propagation velocity is primarily related to *κ*, which proves the validity of the relationship between *κ* and *v*_*φ*_/*c* in Eq. (16).

Then, at the moment in time of Fig. 3(a3–c3), electromagnetic waves are absorbed by the CPML for the most part, except for the area adjacent to the PEC shown as position P4. This phenomenon is the typical dispersion that the electromagnetic field is continuously inducing after the wave front has gone through the grids. If the grids are in a vacuum, the components of the electromagnetic field will increase exponentially. In fact, parameter settings of pa, pb and pc share the same conductivity, which will produce equal damping effects on an electromagnetic wave and dynamic equilibriums on absorption and dispersion. As a result, the amplitude of Hz in the outer CPML can be taken as a key measure of dispersion. Relative to Fig. 3(a3,b3), the amplitude of Hz in Fig. 3(c3) is larger in a broader area, whereas oscillatory waves are closer to the inner CPML. Especially remarkable high-frequency reflection appears in position P4 caused by the outer CPML, where *κ* is larger and the grids are more likely to initiate extensive dispersion. Regardless, the inference of Eq. (16) is proven to be coincidental with the wave field snapshots.

The wave field is similar to Fig. 3(a4–c4) over a long time, which means that the dispersion is simultaneously suppressed and aroused. When we compare the dispersions between Fig. 3(a4) and Fig. 3(b4) in the outer CPML and combine them with the Hz modeling results from pa and pb in Fig. 4(a), it can be seen that the absorption of dispersion in pb is poor, which confirms that the high-frequency electromagnetic waves are indeed produced by dispersion. Therefore, larger *α*_*inn*_ enables the inner CPML to possess a higher dominant absorption spectrum, and the dispersion waves from the outer CPML can thereby be better suppressed. The coverage area of distinct dispersion is extensive in pc because of the large *κ*_*out*_ that forces the inner CPML to start dispersion early. There is some dispersion disturbance in the area whose incidence angle is less than 30° in Fig. 3, and the disturbance is positively correlated with incident angle, which conforms to the regulation of *v*_*φ*_/*c* and *θ* seen in Fig. 1(c).

In sum, Figs 3 and 4 jointly prove that some dispersion is generated in CPML grids when *κ*_*out*_ > 1. The qualitative analysis of optimal parameters shows that small *κ*_*out*_ effectively reduces dispersion at the source, whereas large *α*_*inn*_ partly enhances the inhibitory effect of dispersion. Unfortunately, *α*_*inn*_ is fundamentally unable to eliminate dispersion caused by *κ*_*out*_; otherwise, the CPML method is a promising way to achieve ideal absorption by infinitely increasing *κ*_*out*_.

**Figure 5:**
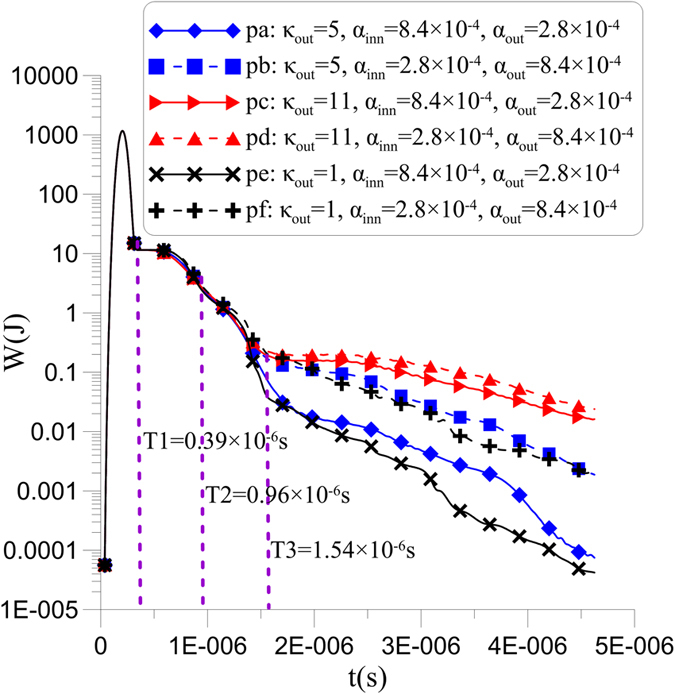
The energy of the wave field in the time domain from six parameters.

Next, we employ the energy of the entire wave field to estimate the dispersion standard and absorption quantity in the CPML (see Fig. 5). Low energy implies the successful absorption of the CPML after the source is turned off (the moment of T1). Within the time that the head waves propagate from the inner CPML to the outer CPML (the period from T1 to T2), the decline rates of energy in pa-pf gradually accelerate and differ little from each other because of the gradually enhanced absorption efficiency in the CPML, whereas the dispersion is not at all prominent. Shortly afterwards, the head waves reflect to the inner CPML (the period from T2 to T3), the energy gradients reach maximum and the curves of pa-pf begin to appear significantly different when electromagnetic waves are mainly absorbed by the outer CPML and different dispersions appear. The energy gradients of pa-pf continuously decrease after the moment of T3; moreover, the curves are significantly distinguishable and comprehensively display the absorption effects from various parameters. Consistent with the conclusions of Figs 3 and 4, large *α*_*inn*_ (such as pa, pc and pe) well restricts the dispersion when *κ* values are the same, small *κ* (such as pe and pf) results in small dispersion when *α*_*inn*_ and *α*_*out*_ are identical, and *α*_*inn*_ contributes less and less to the suppression of dispersion, along with increased *κ*.

Generally speaking, the optimal parameter settings of the CPML should seriously consider dispersion problems caused by *κ*_*out*_. If the dispersion is intolerable, then *κ*_*out*_ = 1. How should the value of *κ*_*out*_ be determined, and does *κ*_*out*_ have relevance to other parameters? To answer these questions, the next section will provide the interpretation employing a low-frequency pulse.

### The strategy for optimal parameters from low-frequency modeling experiments

A severe challenge to low-frequency electromagnetic modeling is the large skin depth. Although CPML can obtain wonderful absorption characteristics for low-frequency electromagnetic waves, it is beyond their abilities to ideally absorb electromagnetic waves through only 8 layers.

Therefore, experiments were conducted to evaluate the absorption effects of the CPML with a low-frequency pulse in a vacuum. We found that, except for the key parameters (*α*_*out*_ and *κ*_*out*_) in the high-frequency experiment, the grid numbers from the source to the innermost CPML (*n*) and the conductivity of the outermost layer (*σ*_*out*_) are significant. In the process of the absorption of low-frequency electromagnetic waves, *α*_*inn*_ and *α*_*out*_ still determine the dominant absorption frequency of the CPML and combined *n* (the grid number from the transmitting coil to the innermost CPML), *κ*_*out*_ and *σ*_*out*_ influence the absorption effect.

**Figure 6:**
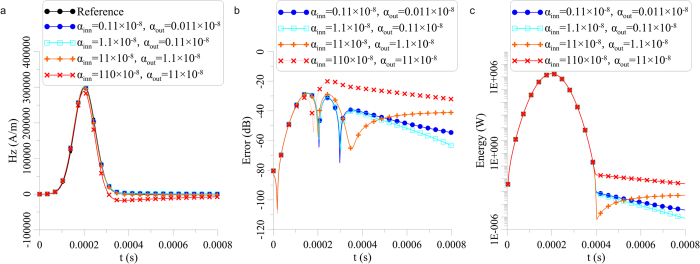
The absorption effects of different *α*_*inn*_ and *α*_*out*_. **(a)** is the contrast of modeling results and analytical solutions, **(b)** is the relative error of modeling changing over time, and **(c)** is the average electromagnetic energy of the inner modeling area at each moment.

First, the requirements of *α*_*inn*_ and *α*_*out*_ with a low-frequency pulse are discussed. Because the basic frequency of a Gauss pulse *f*_*basic*_∈(0,3/*τ*), the energy of a Gauss pulse is maximum when the frequency *f* = 0 and is reduced to 0.048 times the maximum when *f* = 3/*τ* (*τ* is the base width of the Gauss pulse). In this section, *τ* = 0.0004s in Fig. 6(a) results in *f*_*basic*_∈(0,7500)*Hz*. Moreover, the basic degree of freedom of the stretching factor *α*_*basic*_∈(0,120 × 10^−8^) restricts the scope of *α*_*inn*_ and *α*_*out*_ by Eq. (6). CPMLs are generally set as 8 layers due to the restrictions of computer memory. If the space between *α*_*inn*_ and *α*_*out*_ is too large, the intervals of each layer’s *α* will be too far apart to effectively cover the main frequency of the pulse referred to the scheme in Eq. (7). Further optimization is studied to allocate *α*_*inn*_ and *α*_*out*_ for a low-frequency Gauss pulse.

Figure 6 displays the modeling results whose *α*_*inn*_ and *α*_*out*_ truncate (0.011 × 10^−8^, 110 × 10^−8^) into four equidistant sections in exponential coordinates. The modeling areas are similar to the high-frequency experiments. The Hz component in Fig. 6(a) is from a receiving coil located in the same plane (vertical to the Z coordinate) as a transmitting coil 144 m away. The modeling result with *α*_*out*_ = 11 × 10^−8^ differs from the analytical solution of the magnetic dipole Gauss pulse from Eq. (21). It is meaningful to highlight the approximation between modeling results and analytical solutions; accordingly, the relative error^13^ is introduced:


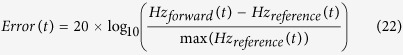


where *Hz*_*forward*_(*t*) is the time-domain Hz of the forward modeling results, *Hz*_*reference*_(*t*) is the time-domain Hz in Eq. (21). According to *Error*(*t*) in Fig. 6(b), the overall relative error is minimum when *α*_*out*_ = 0.11 × 10^−8^ and is maximum when *α*_*out*_ = 0.011 × 10^−8^. Figure 6(c) shows the curves of average energy of the inner modeling area at each moment; the energy attenuation is fastest when *α*_*out*_ = 0.11 × 10^−8^, which is near the characteristic attenuation of electromagnetic energy in a vacuum.

Based on Fig. 6, we confirm that *α*_*inn*_ = 1.1 × 10^−8^ and *α*_*out*_ = 0.11 × 10^−8^ are the optimal parameters for the Gauss pulse. The characteristic frequency of the CPML should be set to *f*_*ay*_∈(0.003/*τ*, 0.03/*τ*). Finally, the general optimal parameters for the Gauss pulse are as follows:


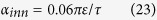



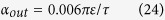


Therefore, the CPML guarantees that the main energy of a low-frequency electromagnetic pulse can be absorbed, taking into account the high-frequency component.

In addition to determining the evaluation of *α*_*inn*_ and *α*_*out*_, Fig. 6 suggests that the dispersive phenomenon is relatively faint in a low-frequency situation. As a consequence, the dispersion from the high-frequency component avoids severe eruption when *α*_*out*_ is small. How then do CPML impacts the absorption effect of relatively low-frequency electromagnetic waves on earth? Although mathematical derivation may be the most rigorous choice, the reality is that hundreds of reflections occur during a single pulse period and in the target area of the crosswell EM. Moreover, a large number of parameters with complex combinations are highly variable in the low-frequency experiments and derivation from formulas for the evaluation of absorption effects encounters discrepancies when compared with realistic modeling. Therefore, this section reports the implementation of 750 modeling experiments whose parameters were reasonably refined locally to extract optimal *n*, *κ*_*out*_ and *σ*_*out*_ from practice.

**Figure 7:**
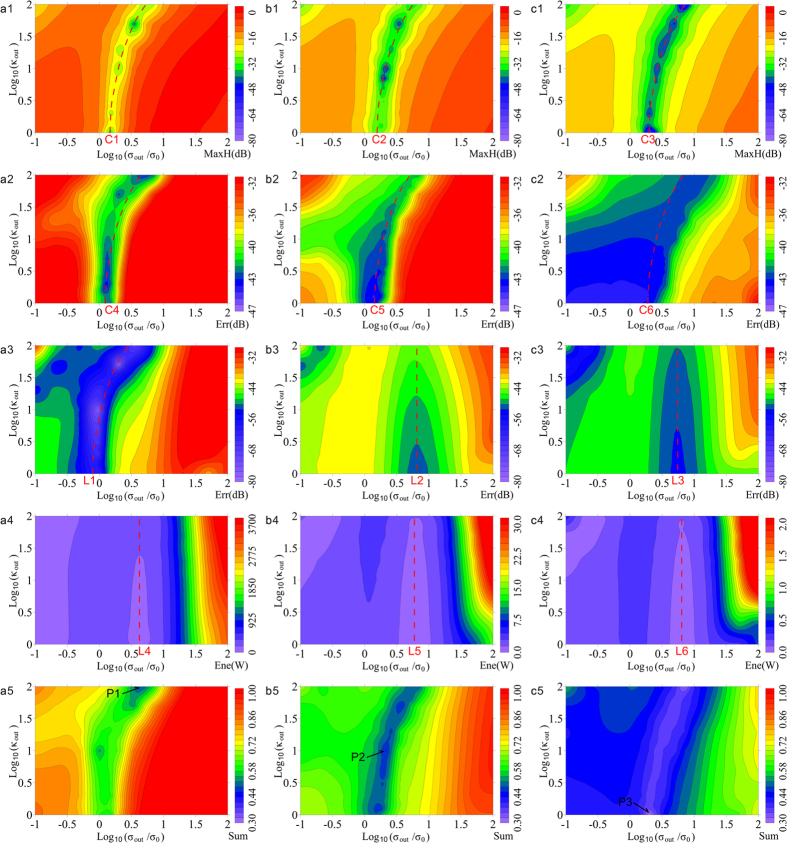
Relationships between the absorption effect of CPML and *n*, *κ*_*out*_ and *σ*_*out*_ a. *n* = 5 in (**a1**)–(**a5**); b. *n* = 11 in (**b1**)–(**b5**); c. *n* = 17 in (**c1**)–(**c5**). 1: (**a1**)–(**c1**) are relative errors of Hz in the receiving coil at the moment of 0.0002 s; 2: (**a2**)–(**c2**) are average relative errors of Hz in the receiving coil during the period 0–0.0004 s; 3: (**a3**)–(**c3**) are average relative errors of Hz in the receiving coil during the period 0.0004–0.0008 s; 4: (**a4**)–(**c4**) are average energies of the inner electromagnetic field during the period 0.0004–0.0008 s; 5: (**a5**)–(**c5**) are optimal indices based on the preceding data. *σ*_0_ = 2.936 × 10^−4^S/m on the X axis.

The grid number *n* from the transmitting coil to the innermost CPML was preset because of the elaborate degree of grids and the restricted size of the modeling area. Therefore, *n* was treated as constant. As is shown in Fig. 7, the combination of *κ*_*out*_ and *σ*_*out*_ influenced the absorption efficiency of CPML for three cases of *n* (named a, b, c). Considering the large range of values of *κ*_*out*_ and *σ*_*out*_, among which the value we are more often concerned with is relatively small, the logarithmic coordinates were implemented in X and Y directions in Fig. 7. Additionally, several significant variables were extracted from Fig. 6: the relative errors at moment 1, the average value of relative errors in period 2 and period 3, and the average value of dumped energy in the fixed inner modeling space in period 4. These variables established corresponding standards 1–4 in Fig. 7 to evaluate the absorption effects from different sizes of modeling space and different CPMLs. Standards 1–3 are based on relative errors whose logarithm operation emphasizes the minimums of modeling error. If the results from a combination of *κ*_*out*_ and *σ*_*out*_ are drawn as cool colors in Fig. 7, this combination is the exact optimal parameter setting we need for a low-frequency Gauss pulse.

Figure 7(a1–c1) and Fig. 7(a2–c2) reflect the influence of relative errors that *κ*_*out*_ and *σ*_*out*_ produce on the primary field at the receiving coil from the perspective of maximums and averages. Logarithmic curves (C1–C6) connect the minimum or cool color areas in each figure. The area of cool color increases and the whole relative error decreases when *n* increases. However, because the amplitude of Hz is inversely proportional to the third power of distance, the increase in *n* causes a decrease of Hz that is reflected from the PEC; therefore, the minimum relative error in Fig. 7(c1,c2) displays the electromagnetic energy’s diffusion. As a result, it was effective to reinforce the absorption of the primary field by expanding the modeling area. Moreover, Fig. 7(a1–c1) show the relative errors of the maximum pulse signal, among which the logarithmic curves move evenly and slightly in the direction of *σ*_*out*_ increase from C1 to C3, and the minimum areas of relative error move markedly in the direction of *κ*_*out*_ decrease. Fig. 7(a2–c2) shows the average relative errors of the receiving signal during the pulse transmission; the areas of minimum relative errors obviously become larger and move in the direction of *κ*_*out*_ decreases. Therefore, the optimal parameters for absorbing the primary field could be found from C1–C6: the optimal *κ*_*out*_ and *σ*_*out*_ both decrease with *n* increase. Therefore, if *n* is small, powerful absorption can be realized by simultaneously enlarging *κ*_*out*_ and *σ*_*out*_ in the CPML; if *n* is large, the energy of electromagnetic waves at the CPML is sufficiently weak to satisfy the absorption requirement merely through *σ*_*out*_, whereas *κ*_*out*_ = 1 is the best choice for dispersion to be entirely removed.

Figure 7(a3–c3) and Fig. 7(a4–c4) were established with a single receiving coil and the entire modeling area, respectively; they reveal the influence of secondary fields from different *κ*_*out*_ and *σ*_*out*._. A difference from the primary field is that the areas of minimum relative error are mostly distributed as isosceles triangles whose vertex angles in each figure can be joined as a straight line (L1–L6). From the perspective of the relative error of the secondary field and the surplus energy of space, the horizontal gradient is much larger than the vertical gradient; therefore, the appropriate choice of *σ*_*out*_ is the key to promoting the modeling precision of the secondary field. Too small *σ*_*out*_ will reduce the absorption performance of the CPML, although too large *σ*_*out*_ will induce a strong current. If *σ*_*out*_ is constant, simply decreasing *κ*_*out*_ plays a minor role in reducing the relative error of the secondary field and suppressing the surplus energy in space. Therefore, the dispersion remains tiny in low-frequency conditions referred to Eq. (16). In contrast with Fig. 7(b3,c3), the shape of relative errors in Fig. 7(a3) is more similar to that in (a1) and (a2). Therefore, *n* ≤ 8 (the modeling results are still similar up to *n* = 8) leads to strong reflection from the PEC and the distribution of relative error after the source turns off remains from the primary field. Additionally, the overall relative error in Fig. 7(b3) is not as small as in Fig. 7(c3), and L3 moves slightly in the direction *σ*_*out*_ increases. The outlines of Fig. 7(a4–c4) are similar, whereas the ranges of color bars differ by more than a factor of 10, which indicates that *n* depresses the secondary field’s energy more than *κ*_*out*_ and *σ*_*out*_. To reduce the relative error of the secondary field, we should first implement *n* > 8, then use a value of *σ*_*out*_ near L6 and keep *κ*_*out*_ small.

Unfortunately, the conclusions of Fig. 7 are inconsistent for the optimal parameters when the sizes or the periods are different. As a solution, the results of standards 1–4 were weighted averaged (see Fig. 7(a5–c5)), which comprehensively considers the error of each moment and each grid. Ultimately, the optimal combinations of *κ*_*out*_ and *σ*_*out*_ are the points P1, P2 and P3 in the conditions of each *n*.

As shown in Table 2, another two groups of optimal parameters were extracted from the modeling areas for which *n* = 8 and *n* = 14, with the addition of the optimal parameters P1, P2 and P3 from Fig. 7. There is some logarithmic relationship between optimal *κ*_*out*_ and *σ*_*out*_ at different *n*. Therefore, *κ*_*out*_ and *σ*_*out*_ both promote the absorption effect of the CPML, although a restriction mechanism exists. The interpretation of this discovery can be seen in Fig. 1: the value of dispersion is linear to *κ*_*out*_ in the CPML, whereas the electromagnetic absorption effect is exponential to *σ*_*out*_^16^; therefore, the logarithmic distribution of optimal parameters precisely corresponds to the conclusion. The key to absorbing the low-frequency electromagnetic wave is suppressing dispersion, and the dispersion caused by *κ*_*out*_ should be absorbed by the corresponding *σ*_*out*_, just as in the high-frequency cases. Then, the combinations of optimal parameters at low-frequency are fitted from Table 2 (the fitting error R^2^ = 0.986.):


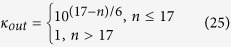



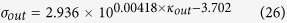


The predicted optimal parameters were calculated from Eqs (25) and (26), whose modeling error-sums were smaller than those of the selected optimal parameters when *n* ≥ 8. This conclusion also verifies the validity of Eqs (25) and (26). To the authors’ knowledge, there is no research that proves that the absorption frequency is related to *κ*_*out*_ or *σ*_*out*_ and the selection standard of optimal parameters can thus be generalized to other frequencies.

The discussions above organize the strategy of optimal parameters in a CPML. First, *α*_*inn*_ and *α*_*out*_ are calculated by Eqs (23) and (24) based on the dominant frequency of the CPML. The maximum grid interval *δ*_max_ is then constrained by the maximum frequency of the pulse and *n* ≥ 8 so that the CPML can efficiently absorb the diffuse primary field. Finally, *κ*_*out*_ and *σ*_*out*_ are calculated with Eqs (25) and (26).

## Discussion

**Figure 8:**
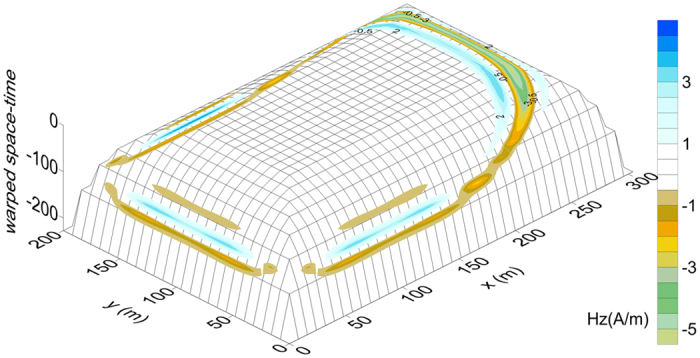
The warped space-time of Fig. 3 (c3) with the CPML.

The absorption of electromagnetic waves in a PML mainly uses the gradually increased *σ* in the absorbing boundary. Essentially, the electromagnetic energy is converted into heat energy. Additionally, the real stretch *κ* in the CPML enlarges the realistic interval of the absorbing boundary, especially the outer layers for which the absorption efficiency is highest. Therefore, the CPML warps the space compared with a uniform grid in a conventional FDTD and the dispersion is an inherent characteristic of the tectonically warped space-time. The existence of this condition is why this research adopted “dispersion” instead of the universal “numerical dispersion.” As we can observe in Fig. 8, the exponential growth of *κ* accompanies the incremental degree of warped space-time. Therefore, the physical relevance of *κ* is the warp degree caused by the artificial substance, which is perhaps analogous to a Schwarzschild black hole^23^, as both a CPML and a Schwarzschild black hole can delay electromagnetic waves^24^. However, CPMLs are not yet able to totally simulate Schwarzschild black holes. Electromagnetic waves in CPMLs propagate in two opposite directions via the reflection of PEC layers before they are able to escape; in fact, CPMLs are not spheres with a Schwarzschild radius. Because of the restriction on grid numbers resulting from the memory limits of computers, space-time in CPMLs warps stepwise with slight interfacial reflection. Moreover, the forward results in CPMLs show that the span of a pulse diminishes and a blue shift appears.

The authors attempted to improve CPMLs by imitating the structure of a Schwarzschild black hole. We set singularities with significant *κ* in the 8 outer corners and guided electromagnetic waves to those corners by modifying *κ*. We moved the maximum *κ* to the outboard second layers so that a groove of space-time may fascinate electromagnetic waves. We attempted to promote renewal equations to high order in the CPML to remedy the dispersion; however, the attempts failed because the grids’ numerical dispersion was small, whereas the warped space-time’s dispersion is large. Nonetheless, we believe that an ingenious scheme will be developed to achieve ideal absorption by simply warping space-time.

## Conclusion

Considering the relatively low transmitting frequency and small grid interval of the crosswell EM, we used the CPML as an absorbing boundary condition of an FDTD and derived the optimal parameters of the CPML to strengthen the advantages of the absorption of low-frequency electromagnetic waves. Theoretical analysis indicated that dispersion in the CPML would inevitably appear when *κ*_*out*_ > 1 and that the dispersion would be severe when *κ*_*out*_ is large and the angle of incidence wave is small, as shown in Fig. 1.

High-frequency experiments proved that dispersion in the CPML exists, especially in the outer layer. Moreover, there is a positive correlation between dispersion and *κ*_*out*_, and it is effective to suppress the high-frequency dispersion waves if *α*_*inn*_ > *α*_*out*_. Further experiments involving low frequencies established the evaluation standard for absorbing boundary conditions. For a Gauss pulse, Eqs (23) and (24) are the optimal parameter settings for *α*_*inn*_ and *α*_*out*_. Finally, no matter how large the *n* values are, there is an optimal choice between the dispersion from *κ*_*out*_ and the absorption effect related to *σ*_*out*_, as shown in Eqs (25) and (26).

## Additional Information

**How to cite this article**: Fang, S. *et al*. Crosswell electromagnetic modeling from impulsive source: Optimization strategy for dispersion suppression in convolutional perfectly matched layer. *Sci. Rep.* **6**, 32613; doi: 10.1038/srep32613 (2016).

## Figures and Tables

**Table 1:** Summary of pivotal parameters in a CPML.

Parameter synthesis	Meaning	Physical/numerical effect	
α (*α*_*ez*_, *α*_*mz*_, *α*_*inn*_, *α*_*out*_)	degrees of freedom	determine the characteristic absorption frequency, suppress dispersion	
*n*	grid number	recast the size of real space	codetermine the absorption efficiency
*σ* (*σ*_*ez*_, *σ*_*mz*_, *σ*_*out*_)	conductivity	transform the EM energy into heat energy	
*κ* (*κ*_*ez*_, *κ*_*mz*_, *κ*_*out*_)	real stretch	dilute the EM energy by warping the space	
*S*_*ez*_	stretching factor	the curl factor of rectangular or stretched coordinates	
*f*, *f*_*a*_	frequency	*f*_*a*_ is the characteristic frequency of absorption	
*a*_*ez*_, *b*_*ez*_, *ψ*_*exy*_, *ψ*_*exz*_	interim parameters	simplify the computational complexity	
*δ*	grid interval	cause the dispersion in conventional grids	

**Table 2:** The statistics of optimal parameters from several values of *n*.

	**Variable**	**Unit**	*n* = 5	*n* = 8	*n* = 11	*n* = 14	*n* = 17
**Selected optimal parameters**	log_10_(σ_out_/σ_0_)	1	0.699	0.544	0.330	0.301	0.301
	log_10_(*κ*_*out*_)	1	2.000	1.699	1.000	0.477	0.000
	σ_out_	10^−4 ^S/m	14.680	10.276	6.283	5.872	5.872
	*κ*_*out*_	1	100.000	50.000	10.000	3.000	1.000
	Error-sum	1	0.508	0.498	0.462	0.380	0.300
**Predicted optimal parameters**	σ_out_	10^−4^ S/m	15.279	7.907	6.421	6.010	5.887
	*κ*_*out*_	1	100.000	31.623	10.000	3.162	1.000
	Error-sum	1	0.579	0.438	0.441	0.362	0.256
